# Sternoclavicular joint septic arthritis with chest wall abscess in a healthy adult: a case report

**DOI:** 10.1186/s13256-016-0856-0

**Published:** 2016-03-26

**Authors:** Yoshihito Tanaka, Hisaaki Kato, Kunihiro Shirai, Yasuhiro Nakajima, Noriaki Yamada, Hideshi Okada, Takahiro Yoshida, Izumi Toyoda, Shinji Ogura

**Affiliations:** Department of Emergency and Disaster Medicine, Gifu University Hospital, 1-1 Yanagido, Gifu, Gifu 501-1194 Japan

**Keywords:** Sternoclavicular joint septic arthritis, Chest wall abscess, Sepsis, Negative pressure wound therapy (NPWT), Hyperbaric oxygen therapy (HBO_2_)

## Abstract

**Background:**

Septic arthritis of the sternoclavicular joint is rare. It can be associated with serious complications such as osteomyelitis, chest wall abscess, and mediastinitis. In this report, we describe a case of an otherwise healthy adult with septic arthritis of the sternoclavicular joint with chest wall abscess.

**Case presentation:**

A 68-year-old Japanese man presented to our hospital complaining of pain and erythema near the right sternoclavicular joint. Despite 1 week of oral antibiotics, his symptoms did not improve. Computed tomography revealed an abscess with air around the right pectoralis major muscle. After being transferred to a tertiary hospital, emergency surgery was performed. Operative findings included necrotic tissue around the right sternoclavicular joint and sternoclavicular joint destruction, which was debrided and packed open. Methicillin-susceptible *Staphylococcus aureus* was identified in blood and wound cultures. Negative pressure wound therapy and hyperbaric oxygen therapy were performed for infection control and wound healing. The patient’s general condition improved, and good granulation tissue developed. The wound was closed using a V-Y flap on hospital day 48. The patient has been free of relapse for 3 years.

**Conclusions:**

Septic arthritis of the sternoclavicular joint is an unusual infection, especially in otherwise healthy adults. Because it is associated with serious complications such as chest wall abscess, prompt diagnosis and appropriate treatment are required.

## Background

The sternoclavicular joint (SCJ) is an unusual site of septic arthritis. It is involved in only 0.5–1.0 % of all joint infections and in less than 0.5 % of joint infections in healthy patients [1–4]. Common risk factors for SCJ septic arthritis include intravenous drug use (21 %), infection at a distant site (15 %), diabetes mellitus (13 %), trauma (12 %), and an infected central venous line (9 %). No risk factors were found in 23 % of patients with SCJ septic arthritis [1]. SCJ septic arthritis may lead to serious complications such as osteomyelitis [5], chest wall abscess [6–8], mediastinitis [9], or myositis [4]. Management of SCJ septic arthritis consists of surgical debridement and intravenous antibiotics [3, 11]. In this report, we describe a case of an otherwise healthy adult with SCJ septic arthritis with chest wall abscess.

## Case presentation

A 68-year-old Japanese man presented to our hospital complaining of pain and erythema near the right SCJ. He had no risk factors for SCJ septic arthritis such as intravenous drug use, infection at a distant site, diabetes mellitus, trauma, laceration, or valvular heart disease. His past medical history and family history were noncontributory. Oral levofloxacin was started. One week later, he went to another hospital for medical care because his symptoms had not improved. Computed tomography (CT) of the chest revealed an abscess with air around the right pectoralis major muscle. He was transferred to a tertiary center for surgical care.

**Fig. 1:**
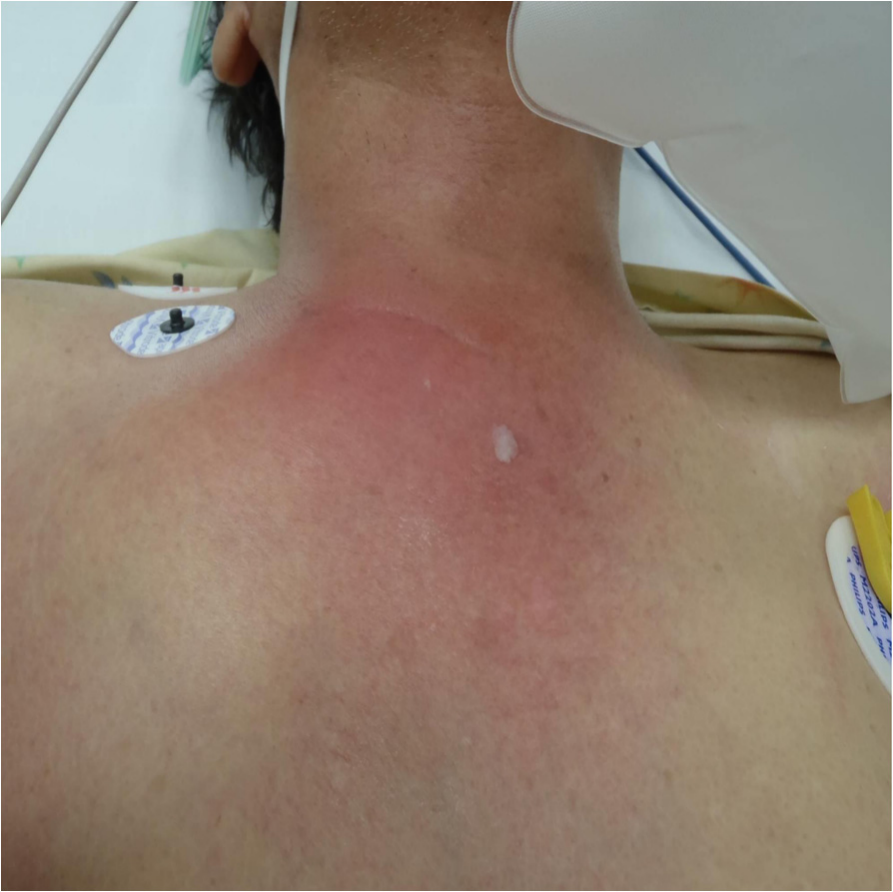
Chest findings on admission. Redness and swelling of skin localizing to the sternoclavicular joint are shown

**Fig. 2:**
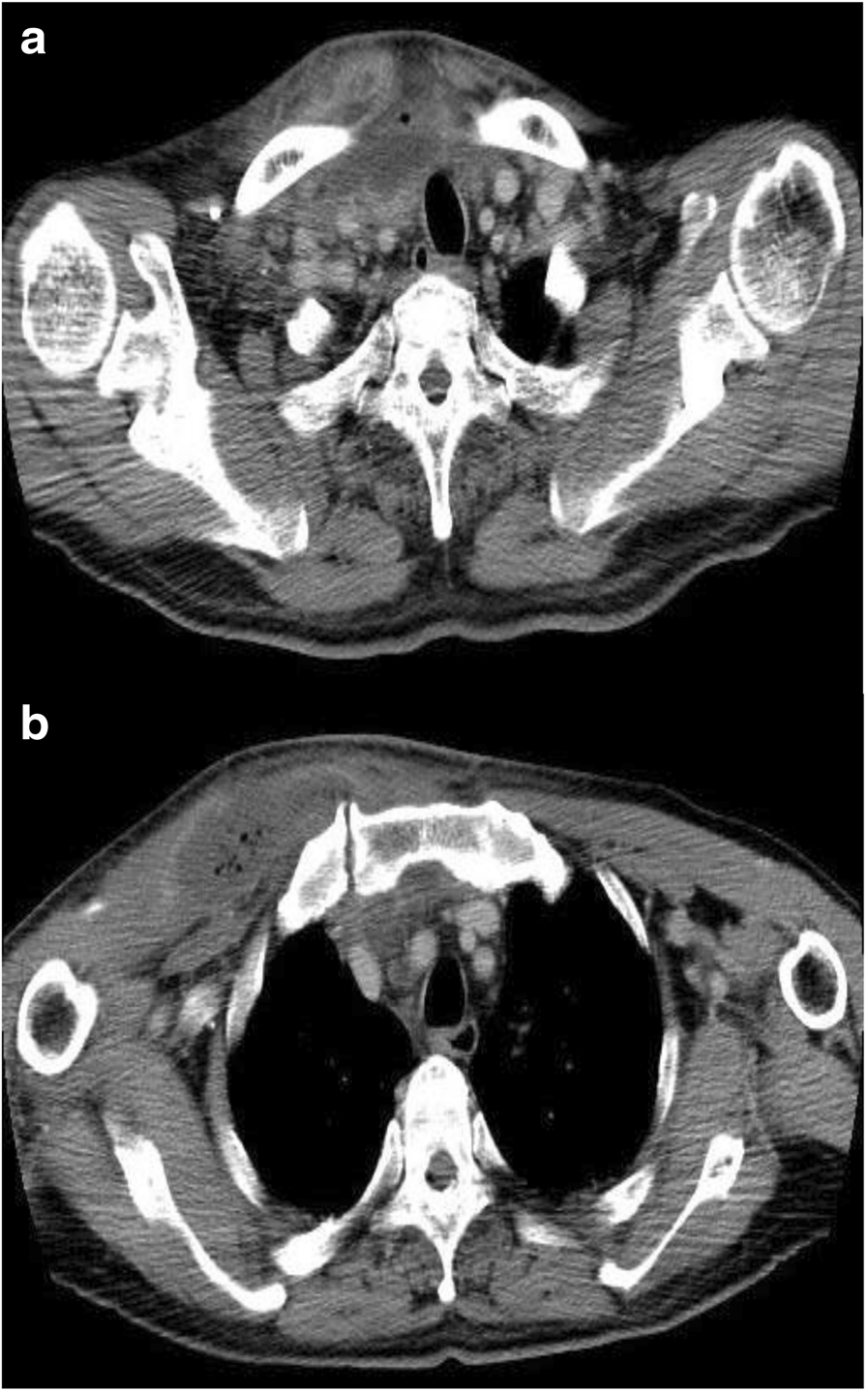
Computed tomographic scans of the chest on admission. Computed tomography of the chest detected an abscess with air located below the thyroid gland and involving the right pectoral major muscle around the right sternoclavicular joint (**a**, **b**), as well as disaggregation of the right sternoclavicular joint (**b**)

His physical examination revealed a body temperature of 39.3 °C, heart rate of 100 beats per minute, and blood pressure of 140/80 mmHg. Signs of inflammation such as redness, swelling, and tenderness over the right SCJ (Fig. 1) were present. His white blood cell count was 14,060/μl, and his C-reactive protein level was 17.5 mg/dl (Table 1). Repeat CT revealed an extensive abscess with air, involving the right pectoralis major muscle, right SCJ, retrosternal region, and right sternocleidomastoid muscle (Fig. 2).

**Table 1:** Laboratory data on admission

Laboratory parameters	Test results
TP	4.2 g/dl
Alb	2.2 g/dl
CPK	127 IU/L
AST	45 IU/L
ALT	28 IU/L
LDH	239 IU/L
ALP	538 IU/L
γ-GTP	81 IU/L
Amy	16 IU/L
Cre	0.80 mg/dl
BUN	15.7 mg/dl
TG	122 mg/dl
T-Chol	85 mg/dl
T-Bil	2.9 mg/dl
D-Bil	1.7 mg/dl
Na^+^	139 mEq/L
K^+^	3.2 mEq/L
Cl^−^	103 mEq/L
Mg	1.9 mg/dl
Ca^2+^	7.1 mg/dl
IP	3.1 mg/dl
CRP	17.5 mg/dl
HbA1c	5.7 %
Arterial blood gas	
FiO_2_	0.6
pH	7.51
pCO_2_	33.0 mmHg
pO_2_	116.0 mmHg
BE	3.2 mmol/L
Lactate	7 mg/dl
WBC	14,060/μl
RBC	356 × 10^4^/μl
Hb	11.0 g/dl
Hct	31.9 %
Plt	14.6 × 10^4^/μl
PT	15.0 seconds
aPTT	33.5 seconds
Fibrinogen	683 mg/dl
FDP	14.2 μg/dl
d-dimer	7.2 μg/dl
AT-III	49 %
Antinuclear antibody	Negative
Rheumatoid factor	Negative

*Alb* albumin, *ALP* alkaline phosphatase, *ALT* alanine aminotransferase, *Amy* amylase, *aPTT* activated partial thromboplastin time, *AST* aspartate aminotransferase, *AT-III* antithrombin III, *BE* base excess, *BUN* blood urea nitrogen, *Ca* ^*2+*^ calcium, *Cl* ^*−*^ chloride, *CPK* creatine phosphokinase, *Cre* creatinine, *CRP* C-reactive protein, *D-Bil* direct bilirubin, *FDP* fibrin degradation products, *FiO*_*2*_ fraction of inspired oxygen, *γ-GTP* γ-glutamyl transferase, *Hb* hemoglobin, *HbA1c* glycated hemoglobin A1c, *Hct* hematocrit, *IP *inorganic phosphorus, *K* ^*+*^ potassium, *LDH* lactate dehydrogenase, *Mg* magnesium, *Na* ^*+*^ sodium, *pCO*_*2*_ partial pressure of carbon dioxide, *Plt* platelets, *pO*_*2*_ partial pressure of oxygen, *PT* prothrombin time, *RBC* red blood cells, *T-Bil* total bilirubin, *T-Chol* total cholesterol, *TG* triglycerides, *TP* total protein, *WBC* white blood cells

**Fig. 3:**
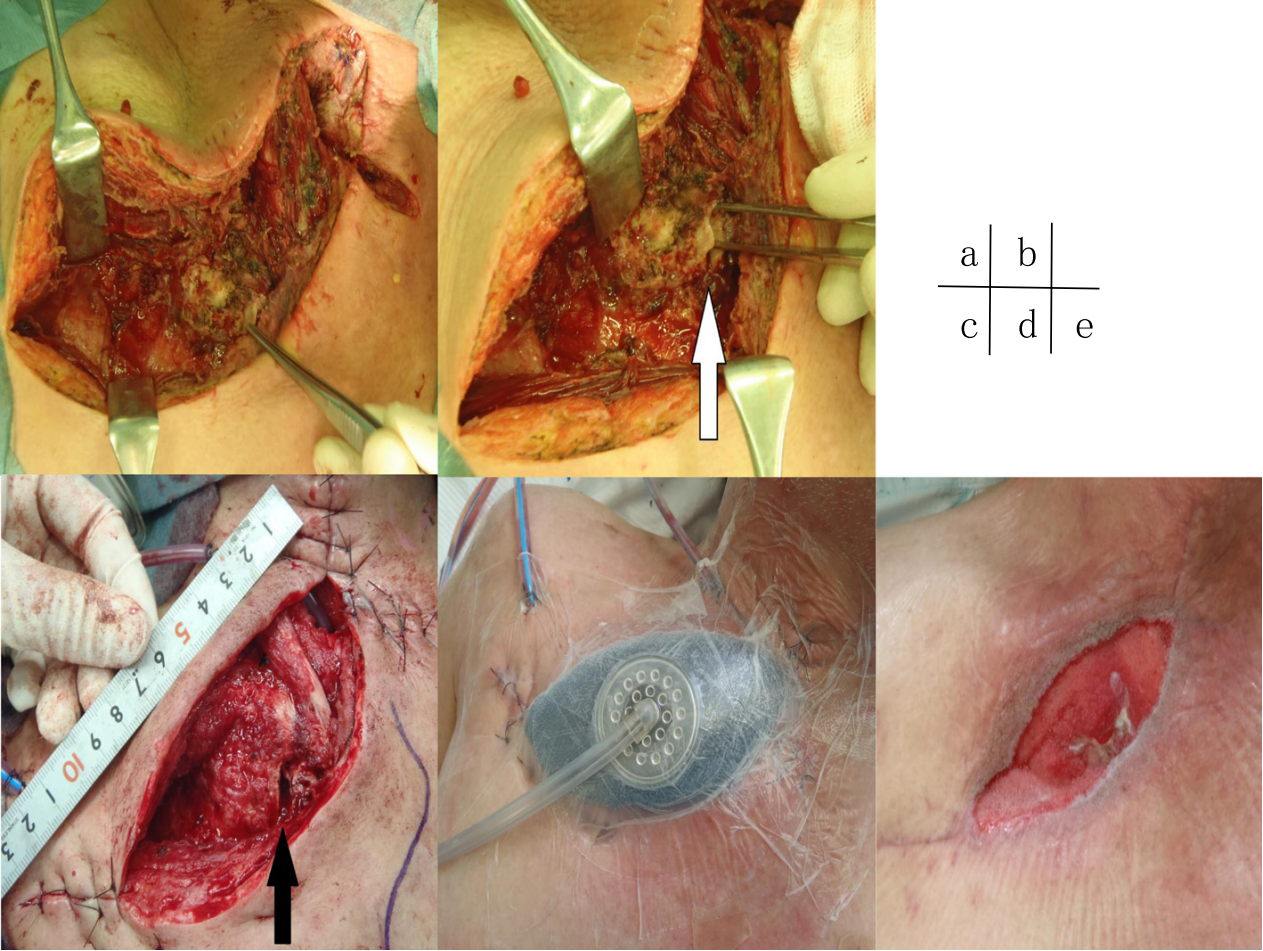
Wound-related findings. Operative findings on the day of admission (**a**, **b**) were necrotizing tissue around the sternoclavicular joint and the joint destruction (*white arrow*). When we debrided residual necrotizing tissue on postoperative day 10, the sternoclavicular joint was still exposed (*black arrow*) (**c**). We introduced negative pressure wound therapy on postoperative day 11 (**d**). On postoperative day 37, good granulation was observed (**e**)

**Fig. 4:**
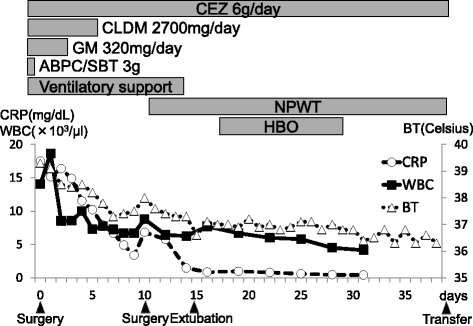
Clinical course. *WBC* white blood cells, *CRP* C-reactive protein, *BT* body temperature, *CEZ* cefazolin, *CLDM* clindamycin, *GM* gentamicin, *ABPC/SBT* ampicillin/sulbactam, *NPWT* negative pressure wound therapy, *HBO* hyperbaric oxygen therapy

Emergency surgical debridement was performed. The skin incision began at the right border of the thyroid and extended to the head of the right clavicle. Operative findings included necrosis of parts of the right pectoralis major and minor muscles and the right SCJ. The patient also had right SCJ destruction. The necrotic pectoralis major and minor muscles and parts of the clavicle and manubrium near the SCJ that had become detached were debrided. The cavity was irrigated and packed open (Fig. 3). An initial Gram stain revealed gram-positive cocci. Ampicillin/sulbactam, which was given preoperatively, was changed to cefazolin (6 g/day), gentamicin (320 mg/day), and clindamycin (2700 mg/day) after surgery. On hospital day 6, methicillin-susceptible *Staphylococcus aureus* was cultured from blood and wound specimens. Antibacterial therapy was tapered to intravenous cefazolin and continued for 6 weeks to treat osteomyelitis (Fig. 4). On postoperative day 10, residual necrotic tissue was debrided, and part of the wound edges was sutured together. After surgery, negative pressure wound therapy (NPWT) and hyperbaric oxygen therapy were performed for infection control and wound healing. The patient’s general condition improved, and there was good granulation tissue formation. He was transferred to his hometown hospital, and the wound was closed using a V-Y flap on hospital day 48. The patient has been free of relapse for 3 years.

## Discussion

Septic arthritis of the SCJ is rare, involving only 0.5–1.0 % of all joint infections. It occurs in less than 0.5 % of otherwise healthy adults [1–4]. It may cause serious complications such as osteomyelitis [5], chest wall abscess [6–8], mediastinitis [9], or myositis [4], with an increased risk of irreversible tissue damage and possibly death [4, 10].

Among 180 cases of SCJ septic arthritis, Ross *et al.* identified the following predisposing risk factors: intravenous drug use (21 %), distant site of infection (15 %), diabetes mellitus (13 %), trauma (12 %), and infected central venous line (9 %). No risk factor was found in 23 % of the patients [1]. The route of infection is often unknown, especially in otherwise healthy patients [3].

The clinical signs of SCJ septic arthritis are chest pain localizing to the SCJ (78 %), fever (65 %), and shoulder pain (24 %). SCJ septic arthritis infrequently presents with neck pain (2 %) [1, 2]. Therefore, septic arthritis should always be considered in the differential diagnosis of chest and neck pain and fever.

CT or magnetic resonance imaging (MRI) should be performed routinely in all cases of SCJ arthritis [1] to determine the severity of the infection and guide the surgical strategy [12–14]. CT and MRI can demonstrate joint subluxation, joint destruction, periarticular inflammation, and SCJ abscess.

The rate of positive cultures with needle aspiration is 77 %, compared with 36 % with surgical debridement and 13 % with blood culture [1]. *S. aureus* is responsible for 49 % of culture-positive infections, *Pseudomonas aeruginosa* for 10 %, *Brucella melitensis* for 7 %, and *Escherichia coli* for 5 %. *Mycobacterium tuberculosis* infection occurs infrequently [1].

Management of SCJ septic arthritis consists of surgical debridement and intravenous antibiotics [3, 11]. Song *et al.* reported that complete SCJ resection and pectoralis flap closure resulted in no recurrences among patients with SCJ septic arthritis, while debridement and antibiotic therapy alone were associated with recurrence in five of six patients [12]. In our patient, because SCJ infection was limited to a small area, partial debridement was performed instead of complete resection. The cavity after debridement was relatively small, and NPWT was performed for wound granulation. After methicillin-susceptible *S. aureus* was identified in blood and wound cultures, broad-spectrum intravenous antimicrobial therapy was switched to intravenous cefazolin, which was continued for 6 weeks to treat osteomyelitis.

## Conclusions

SCJ septic arthritis is a rare infection, especially in healthy adults. Because SCJ septic arthritis is associated with a risk of serious complications such as chest wall abscess, prompt diagnosis and appropriate surgical and antibacterial treatment are required.

## Consent

Written informed consent was obtained from the patient for publication of this case report and any accompanying images. A copy of the written consent is available for review by the Editor-in-Chief of this journal.

## Abbreviations

ABPC/SBT: ampicillin/sulbactam
Alb: albumin
ALP: alkaline phosphatase
ALT: alanine aminotransferase
Amy: amylase
aPTT: activated partial thromboplastin time
AST: aspartate aminotransferase
AT-III: antithrombin III
BE: base excess
BT: body temperature
BUN: blood urea nitrogen
Ca^2+^: calcium
CEZ: cefazolin
Cl^−^: chloride
CLDM: clindamycin
CPK: creatine phosphokinase
Cre: creatinine
CRP: C-reactive protein
CT: computed tomography
D-Bil: direct bilirubin
FDP: fibrin degradation products
FiO_2_: fraction of inspired oxygen
GM: gentamicin
γ-GTP: γ-glutamyl transferase
Hb: hemoglobin
HbA1c: glycated hemoglobin A1c
HBO: hyperbaric oxygen therapy
Hct: hematocrit
IP: inorganic phosphorus
K^+^: potassium
LDH: lactate dehydrogenase
Mg: magnesium
MRI: magnetic resonance imaging
Na^+^: sodium
NPWT: negative pressure wound therapy
pCO_2_: partial pressure of carbon dioxide
Plt: platelets
pO_2_: partial pressure of oxygen
PT: prothrombin time
RBC: red blood cells
SCJ: sternoclavicular joint
T-Bil: total bilirubin
T-Chol: total cholesterol
TG: triglycerides
TP: total protein
WBC: white blood cells
